# Evidence uptake is only part of the process: Stakeholders’ insights on WHO treatment guideline recommendation processes for radical cure of *P*. *vivax* malaria

**DOI:** 10.1371/journal.pgph.0002990

**Published:** 2024-03-14

**Authors:** Varunika S. H. Ruwanpura, Koen Peeters Grietens, Ric N. Price, Kamala Thriemer

**Affiliations:** 1 Global Health Division, Menzies School of Health Research and Charles Darwin University, Darwin, Australia; 2 Institute of Tropical Medicine, Antwerp, Belgium; 3 School of Tropical Medicine and Global Health, Nagasaki University, Nagasaki, Japan; 4 Mahidol-Oxford Tropical Medicine Research Unit (MORU), Faculty of Tropical Medicine, Mahidol University, Bangkok, Thailand; 5 Nuffield Department of Clinical Medicine, Centre for Tropical Medicine and Global Health, University of Oxford, Oxford, United Kingdom; Indian Council of Medical Research - National Institute of Epidemiology, INDIA

## Abstract

Health policy processes should be evidence-informed, transparent and timely, but these processes are often unclear to stakeholders outside the immediate policymaking environment. We spoke to 36 international malaria stakeholders to gain insights on the processes involved in the World Health Organization’s Global Malaria Programme’s recommendations for their treatment guidelines of *P*. *vivax* malaria. Four key themes which drew on the 3i policy framework and Shiffman’s four factors that influence global and national policymaking were identified to understand these processes. Triggers for policy change and change prioritisation, evidence types that inform policy, effects of funding on decision-making processes, and transparency and communication of these processes to external stakeholders. Results indicate that more clarity is needed on what triggers global malaria policy change processes, a clearer justification of evidence types used to inform policymaking, better understanding of the impact of the WHO’s funding model on policymaking and further transparency and improved communication of these processes to external stakeholders is also needed. We suggest that global malaria policymaking could be improved by using the following strategies: ensuring that identified triggers actually initiate the policy change process, expediting decision-making timelines by developing a priority framework for assessing new evidence, adopting suitable frameworks to assess contextual evidence, and increasing the transparency of the role of non-state funders in policy decision-making processes and when publishing new recommendations.

## Introduction

Policy processes for public health responses receive less attention than the new treatments or vaccines themselves. However inefficient processes that enable the use of new tools result in significant delays in patients receiving access to better care. Policy development should be evidence-informed, transparent and timely for improved health outcomes [1], however many global and national health policymaking processes remain ‘black boxes’ lacking the transparency needed to understand the drivers, protocols and specific processes that determine how policy is made [2–6].

In this paper, we focus on malaria policy processes in the context of the World Health Organization’s (WHO) Global Malaria Technical Strategy of achieving malaria elimination in 35 countries by 2030 [7]. Countries close to elimination and endemic for the two most important malaria species, *Plasmodium falciparum* and *Plasmodium vivax (P*. *vivax)*, are facing particular challenges. Vivax malaria is more difficult to eliminate because of its dormant liver forms (hypnozoites) that can cause recurrent disease (relapse) without reinfection from a mosquito bite. In some regions up to 80% of acute episodes of *P*. *vivax* malaria are caused by relapse rather than new infections [8, 9]. Radical cure, the treatment of both the blood-stage of the parasite and the dormant liver form, can prevent relapse and is therefore critical to malaria elimination. The 8-aminoquinolone compounds primaquine and tafenoquine are the only available drugs that remove liver forms from the human host but can cause severe haemolysis (destruction of red blood cells) in patients with low levels of the enzyme Glucose-6-Phosphate Dehydrogenase Deficiency (G6PD). The WHO therefore recommends that all patients are tested for G6PD deficiency prior to treatment [10]. Until recently, G6PD testing was restricted to higher-level laboratories even though the greatest burden of *P*. *vivax* is in remote areas at the periphery of national health systems where access to diagnosis and treatment is limited.

The complexity of *P*. *vivax* treatment results in low global coverage of treatment fuelled by several factors. Firstly, health workers are often reluctant to treat patients without knowing their G6PD status anticipating that administering primaquine, the currently recommended 8-aminoquinoline, will cause haemolysis [11, 12]. Secondly, effectiveness of radical cure treatment is limited by low treatment adherence to multi-day treatments [13–15]. Thirdly, currently recommended dosing regimens may provide suboptimal anti-relapse efficacy in many regions [16].

New tools are now available to address these challenges, including a near-patient G6PD analyser [17, 18], single-dose radical cure with tafenoquine [19, 20] and shorter, higher courses of primaquine [20–23] and their implementation could accelerate malaria elimination. Some countries have less than four years to enter WHO certification to be eligible for elimination in 2030; the timeliness of global and national policy processes are therefore critical.

Malaria is often viewed as a highly technical problem that can be addressed with technical solutions alone [24–26]. Yet non-technical factors that impact if and how evidence is used in policy development are often not scrutinised and under-appreciated. Examples include individual, organisational, political and economic factors. A key gap in our understanding is how these factors influence current WHO policy processes [27] and how they affect country needs in the context of malaria elimination. This paper explores how clinical evidence is used and adopted into policy at global level and whether this process delivers timely, high-quality, and evidence-informed guidance that can be readily adapted by national malaria control programs to suit their local settings.

## Methods

### Overview of study design

Our study used in-depth interviews to explore the following a priori determined themes, a) how and what types of evidence informs global malaria policymaking and b) what other factors influence global malaria policymaking. COREQ guidelines on participant selection, interview guide preparation, note taking and analysis were followed [28].

### Analytical frame

A previous study identified key barriers and enablers of uptake of evidence into health policy as organisational, communication, timing, individual and context [29]. This framework was adapted and used to first categorise data by the factors that may impact the GMP’s policymaking processes. Clar et al. [29] considered evidence as part of “organisational” factors however we considered evidence separately as it is key to the GMP’s policy decision-making [30, 31]. Based on this adaption of Clar et al’s [29] theory and emerging themes from data analysis, we identified six main factors that could impact global policy decision-making for *P*. *vivax* malaria:

1) **Available evidence**–new or emerging on *P*. *vivax* malaria radical cure options and diagnostic tools. 2) **Organisational**–new streamlined work processes between the GMP and the WHO prequalification unit, WHO’s funding model and its overall organisational aims. 3) **Communication**–within the GMP and the prequalification unit, between the GMP and the prequalification unit, and with external stakeholders. 4) **Timing**–of new *P*. *vivax* recommendations in relation to countries’ needs. 5) **Individual**–how individual views at the GMP and the prequalification unit may impact policy decision-making. 6) **Context** of *P*. *vivax* malaria radical cure policy decision-making: the GMP’s interpretation of global and regional disease burden, endemic countries’ needs and its priority for recommending new radical cure options.

Thereafter, categories described in the “3i policy framework” [32, 33] and Shiffman’s “four factors influencing global and national health policy” [34] were used to further understand how the identified factors could influence the policymaking process. Collectively these two related policy frames provide a strong basis for interpreting stakeholder hierarchies and relationships, and how these can influence policymaking. The 3i framework assumes that policy change processes are influenced by three factors: actors’ interests, ideas transmitted via stakeholder networks and institutions that make decisions. Similarly, Shiffman’s identified four factors that influence global and national health policymaking are actor power, ideas, context, and issue characteristics. Global health governance’s complexity makes it difficult to adopt one framework to guide global health policy analysis, supporting the adoption and synthesis of compatible frames for analysis [35]. Thematic categories described in the 3i and Shiffman’s frames were used to understand how the six factors identified by us may impact global malaria policymaking (S1 Appendix).

### Sampling technique

Purposive sampling was used to identify first round interviewees who could provide expert insights on global malaria and wider health policy processes. Interviewees were selected from the WHO, international non-governmental, funding and research organisations involved in malaria efforts, pharmaceutical companies, and global health policy experts. Interviewees were contacted individually via the research team’s professional contacts. Theoretical sampling [36] was then used to identify more respondents based on first respondents’ recommendations, the research teams’ contacts and how the analysis was developing (including representatives of government research organisations and independent malaria researchers).

### Data collection

A total of 43 semi-structured interviews were conducted with 36 global malaria stakeholders. In this paper, we only distinguish between stakeholders internal and external to the WHO to ensure anonymity. Respondents were de-identified and referred to as R1 to R36 before article submission. Question guides were developed around the categories defined in the adapted analytical frame. The guide was adapted for each interviewee based on respondent’s expertise, and in keeping with themes arising from previous interviews. VR developed all question guides and KT & CAL reviewed these before interviews to verify if lines of questioning matched research aims. In-depth interviews, of between 40–60 minutes duration, were conducted from December 16, 2020, to December 12, 2022. Due to COVID-19 travel restrictions, interviews were conducted via Zoom [37], with further follow-up interviews conducted with selected respondents to elicit further information and verify initial responses. In some cases, up to three follow up interviews were conducted with a respondent to verify developing themes. Informed consent was obtained before each interview via either a signed participant information sheet or verbal consent was recorded on the interview transcript (with respondent’s permission) when that was more convenient for the respondent. Ethics approval was obtained for both written and verbal consent procedures.

### Data analysis

**Fig 1:**
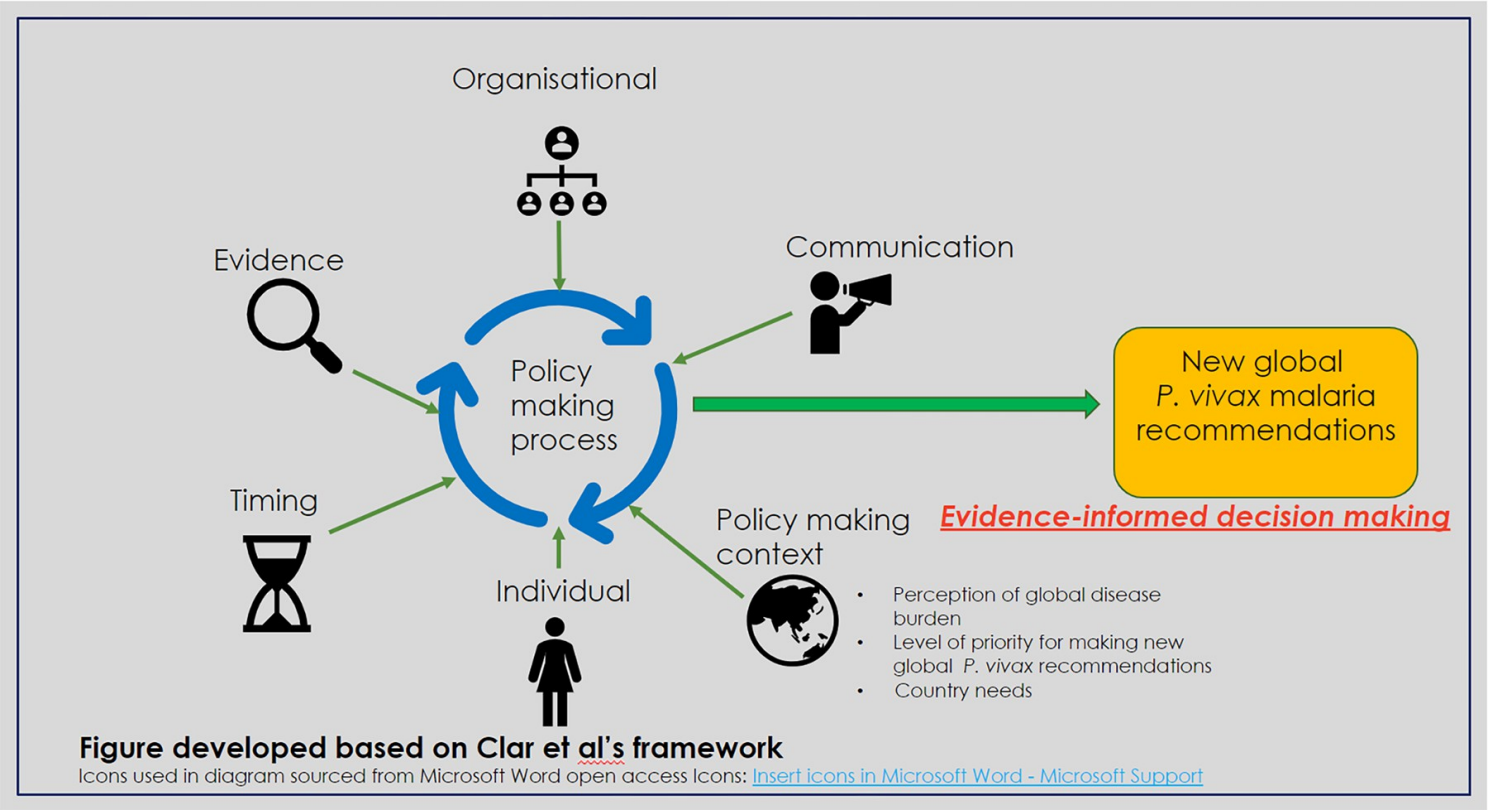
Factors that could influence introduction of new global *P*. *vivax* malaria guidelines.

Interviews were recorded, transcribed verbatim using Otter.ai [38], edited for accuracy and uploaded to NVivo12Plus for analysis [39]. Reflexive memos were written after interviews with each respondent to identify emerging themes and understand the primary researcher’s (VR) own positioning. Interview transcripts were first coded by identified barriers and enablers (Fig 1). A second cycle coding was then done to interpret how these barriers and enablers related to categories described in the 3i framework and Shiffman’s framework. For each theme discussed in results, the adopted frameworks were used to identify factors articulated by respondents as influencing the GMP’s policy decision-making processes.

### Literature used to inform questions guides and analysis

Relevant WHO documents (S2 Appendix) were used to i) inform developed question guides, ii) verify responses obtained at interviews with documented processes, iii) during intermediate interview data analysis to identify gaps needing exploration in further interviews. These included WHO’s handbook for guideline development [27], its global malaria treatment and elimination recommendations and strategy [40–43], WHO’s horizon scanning processes for anticipating new products in the manufacturing pipeline (S3 Appendix) and policy process and supporting documents provided by respondents (S4–S6 Appendices).

### Definitions

“Contextual evidence” was used as an umbrella term to describe feasibility, acceptability and cost-effectiveness evidence.

### Ethics

Respondents were e-mailed a participant information sheet and asked to provide written or verbal consent before interview. Verbal consent was recorded via Zoom [37]. The study was reviewed and approved by the Office of Research and Innovations, Ethics, Charles Darwin University, Australia (H19081) and has received a waiver of full ethical review by the Human Research Ethics Committee of the Australian Northern Territory.

#### Ethics approval and consent to participate

For the qualitative interviews, the participant information sheet was provided via an invitation email. Before commencing interviews, written pre-approved informed consent and verbal informed consent to continue and to record the interview were obtained. All research was done in in accordance with the Declaration of Helsinki and the study protocol was approved by the Human Research Ethics Committee of the Northern Territory Department of Health and Menzies School of Health Research (HREC #H19081).

### Study context

Within the WHO, two departments play key roles in developing global malaria guidelines − the Global Malaria Programme (GMP) and the WHO prequalification unit. These internal processes are important to understand because most external stakeholders depend on the GMP’s global recommendations to fund or introduce antimalarial policy in countries.

### WHO guideline development processes

The guideline development processes are fully explained in the WHO handbook for guideline development [27]. It stipulates that any WHO treatment recommendation is informed by “rigorous adherence to the systematic use of evidence” as per the WHO’s Twelfth General Programme of Work [27]. The WHO Quality Assurance and Norms (QNS) department is also consulted by WHO disease programs to ensure guideline development processes align with WHO standards [44]. WHO recommendations are advisory for countries to interpret and adapt [45].

The GMP’s malaria policy decision-making is overseen by a Guideline Steering Group (GSG) that appoints Guidelines Development Groups (GDGs), comprising external consultants to develop global recommendations [45]. The GSG provides a draft PICO (population, intervention, control, outcome) question guided by the GRC to a GDG. A GDG can refine the PICO question, by group consensus led by its co-chairs, for appropriate evidence inclusion criteria and critical outcomes expected by the GSG [45]. GDGs base their recommendations on evidence reviews presented to them by WHO-appointed Systematic Review Groups (SRGs). SRGs conduct a Cochrane systematic review or draw on previously published systematic reviews to report results to a GDG [45]. GDGs use these results and a GRADE evidence-to-decision-making table [27] to design recommendations. The GDG makes a recommendation to the GMP that is peer reviewed by an External Review Group [45]. External Review Group members are often selected from the WHO’s Malaria Policy Advisory Group (MPAG), formerly known as the Malaria Policy Advisory Committee (MPAC), made up of external consultants with experience in malaria research and treatment [45]. Finally, the WHO’s Guideline Review Committee–that oversees guidelines development–approves the recommendation and the GMP issues *strong* or *conditional* guidelines [27] on the strength of reviewed evidence based on GRADE criteria [45]. The role of the Guideline Review Committee is to provide final oversight over updated guidelines and this committee refers to the decision-making groups below it when making this final approval.

Published guidelines provide details on the *strength of the evidence* supporting the recommendation−which is categorised as strong or conditionally for or against a new intervention [10]. Published malaria guidelines also include practical information or “guidance” that aims to assist countries to adapt recommendations to their contexts. Guidance is based on the WHO guideline development handbook’s stipulation that recommendations must be implementable and adapted to local contexts [27].

### Prequalification unit processes

The WHO’s prequalification unit operates a drugs and diagnostics prequalification programme that functions similarly to a Stringent Regulatory Authority (SRA) [46] and works in parallel with the GMP’s processes [45]. This prequalification process provides countries with quality assurance of WHO-recommended drugs and tools for malaria control. This unit also uses a horizon scanning process to search for any new tools in development that could enter the prequalification pipeline and be recommended in the future [47].

### Changes in process

The main differences between current and previous WHO processes for developing malaria guidelines include: 1) drugs and diagnostic tools’ prequalification processes and the GMP’s policy recommendation processes now working parallelly (tafenoquine is the first drug being assessed using this parallel process), 2) stronger reliance on GDG recommendations for developing malaria guidelines, 3) introduction of an editorial group into the GMP’s guideline recommendation process (the first WHO disease program to do this) and, 4) the increasing role of the WHO Quality Assurance and Norms department in standardising WHO’s policies and processes across disease departments (S7 Appendix).

## Results

**Fig 2:**
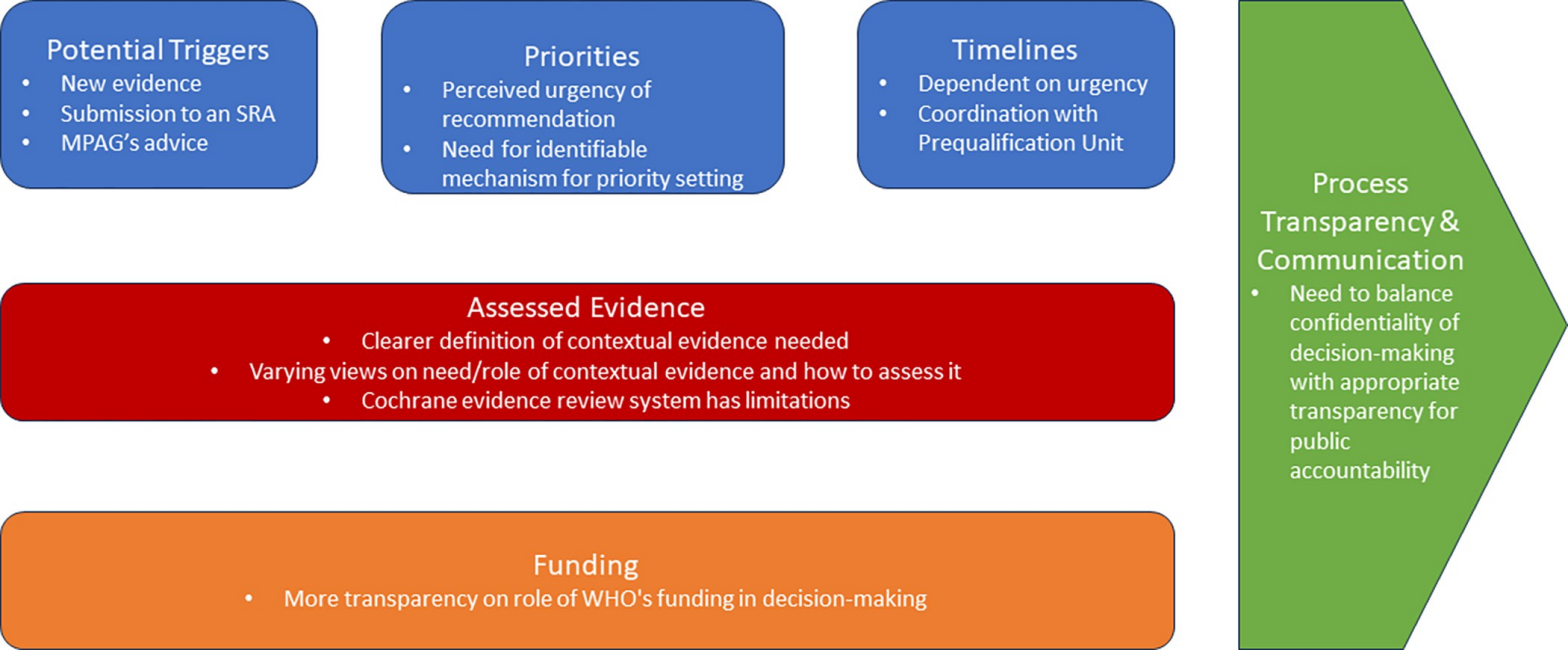
Emerging themes identified regarding the WHO malaria policy pathway.

We present our main results by four themes: i) triggers for change, prioritisation, and timeliness of processes, ii) evidence types and role of contextual evidence in informing guidelines, iii) moderating effect of funding on decision-making processes, and iv) process transparency and communication (Fig 2).

### Triggers for change, prioritisation, and timeliness of processes

#### Triggers for changing current WHO malaria guidelines

Respondents described specific triggers to initiate treatment guidelines changes such as, availability of new evidence indicating that current guidelines are “out-of-date or not consistent with all available evidence” (R28, R13), submission of a dossier for a new tool to a SRA (R17, R18), or policy change initiation on the MPAG’s advice (R27).

According to external respondents, WHO prequalification processes for tafenoquine and the quantitative G6PD analyser should be triggered by dossier submission to SRAs, and the GMP’s guideline revision process by new evidence on high dose primaquine treatment (R26, R28). However, according to WHO respondents the process of working within a new streamlined system for the GMP and the prequalification unit and COVID-related issues have contributed to delays; new recommendations for primaquine based treatments were only released in November 2022 [48]. WHO respondents identified setbacks due to, a) delays in assessing new evidence on treating *P*. *vivax* as staff worked on the WHO’s COVID-19 response (R15), b) prequalification unit needing to assess the quantitative G6PD test’s dossier, inspect manufacturers’ sites once GMP makes a recommendation on this test and subsequently identify laboratories suitable for evaluating the performance of this test (R17, R18) and, c) COVID further delaying these prequalification unit processes especially manufacturing site inspections (R17, R18).

#### Prioritisation of guideline change and prequalification of new tools

Once the policy change process is triggered, guideline development is prioritised by the GMP based on its assessment of the urgency for new recommendations (R27, R13, R14). Views differed on whether individual GMP actors’ power and interests impact overall priority setting and progress of guideline development. No respondent identified an agreed mechanism by which the GMP’s guideline development is prioritised. Some conceded that senior staff may impact development of new guidelines if they delay approving a stage in the decision-making process (R19, R13, R14). Previous GDG recommendations were occasionally delayed when WHO staff’s views could not be readily reconciled with GDG recommendations (R13, R27, R28).

“…it [leadership] has the potential to affect the processes… it might affect the speed, might affect the priority, it might affect how fast it goes.” (R14).

“There was no absolute consensus, particularly among some of the old hands that like to hold countries tight in their fist.” (R27)

A respondent from another WHO department said that there was no influence of individual actors in their department’s work processes and decision-making:

“…for us, it’s not, that’s not the case. It’s a quality management system, whoever comes [into leadership] will have to operate within that quality management system.” (R16)

A key aim of the WHO QNS department is to ensure that such discrepancies in processes and decision-making across the WHO are avoided (R34). For example, in 2023, a new system tracking movement of products through the different stages of the prequalification pipeline is planned with the QNS department’s assistance (R34). External respondents said that the pace of global malaria guideline change also depends on when a topic is introduced to a GDG by the GMP, i.e., when the GMP sets priority for discussing a malaria topic to change a specific malaria guideline and establishes a GDG (R28, R31).

#### Timeliness of global malaria guideline recommendation processes

Timeliness of issuing new guidelines depends on the GMP’s perception of its urgency for countries, when a topic is introduced to a GDG, assessment of the strength of emerging new evidence and need to coordinate with prequalification unit processes (R16, R18, R29, R31, R33). Before the formal guideline development process is initiated, there is often a period when new global evidence is emerging and when GDGs are being formed to assess evidence which also takes an undefined amount of time (R12, R13, R28).

While external stakeholders expressed urgency for new *P*. *vivax* malaria guidelines (R23, R24, R25, R32), WHO respondents’ (R12, R13, R14, R15, R16, R17) emphasised the need for carefully assessing the overall public health benefit of new tools and the feasability of new diagnositcs in real world settings:

“…we are not ready yet to take tafenoquine through the guideline process. Because there is a challenge. If we do that, we make a recommendation that tafenoquine can be used for vivax relapse treatment and all of that to prevent relapse and all of that, we have a practical problem. The recommendation would be, it has to be for places with individuals with G6PD levels of certain percentage, and we don’t have a diagnostic tool yet. So, it is not a problem with tafenoquine. It’s a problem of looking at it from a public health perspective. Why do you want to make a recommendation that we know…it’s difficult to implement?” (R14)

Overall, the need for caution prevailed among those involved in the guideline development:

“But it’s a trade-off, right? Do you want to put a bad recommendation out earlier? That’s the worst thing from a ‘do no harm’ perspective.” (R34)

Most external stakeholders viewed the WHO’s malaria guideline recommendation processes as too slow, currently taking over a year and sometimes several years. This was attributed partly to considering “too much and too many types of evidence”, conservative decision-making and differing opinions within the GMP on the best process by which to make new guidelines (R2, R4, R6, R7, R8, R11, R24, R23, R25):

“I think sometimes the WHO is hesitating. And that’s why we still don’t have that recommendation [on *P*. *vivax* radical cure options], because they want to go that one step further. They’re like, ’we need to be very careful, we need to make sure that countries don’t do…’. So, there’s a lot of like, don’t do all these things. Instead of saying here are all the options and you decide on how you’re going to handle this.” (R24)

“I think, it’s a well-intended process, but has been desperately and I think dangerously slow.” (R28)

“WHO is already incredibly too slow. And now that they have this process [new streamlining], it seems like they’ve inserted a three-to-five-month delay in policy.” (R32)

WHO respondents estimated guideline development timelines to be between six (rapid response guidelines) to 18 months with most suggesting the longer timeframe (R13, R15, R27). However, external respondents estimated the timeframe as up to seven years in some cases (R10, R31, R33).

“…there’s no guarantee from WHO that, you know, generically, if a product is registered, or a phase three trial completed today, or the data is made available today, then a recommendation, either in favour or against would be made in a certain timeframe.” (R12)

### Evidence types and the role of contextual evidence in informing guidelines

Evidence types informing the GMP’s guidelines are primarily systematic reviews of evidence from randomised control trials (RCTs) using a Cochrane review approach, and evidence from non-randomised studies when RCT evidence is unavailable (R13, R28). However, the Cochrane system that SRGs use to review all evidence is mainly designed to assess RCT trials:

“And there was quite a lot of discussion [in GDGs] around how to deal with issues where important data was generated outside of randomised controlled trials, because Cochrane was pretty much set up believing in randomised control trials as the only way of generating evidence. So, they weren’t great at including evidence from cohort event monitoring studies, single arm studies.” (R28)

Available contextual evidence on feasibility, acceptability, cost effectiveness and implementation is also considered as WHO’s handbook for guideline development states that whether guidelines “can be implemented in and adapted to local settings and contexts” [27] should be considered (R28, R29, R30). The handbook is considered “the bible” for guideline decision-making by WHO staff (R12, R13).

What comprises contextual evidence is undefined, but it may determine the strength of the recommendation (R13).

“…any data on cost effectiveness related to these [G6PD] tests will be considered in the guideline development process. But it really, it really impacts the strength of the recommendations…it doesn’t make or break the recommendations.” (R15).

NMP members’ (who sit on a GDG) views on feasibility of the guideline being developed are conveyed to the GMP (R13) but how much these views inform final guidelines is unclear to external stakeholders (R2, R25, R26, R31).

While writing this paper, a GDG updated several malaria recommendations including primaquine recommendation for vivax malaria and treatment in the first trimester of pregnancy [48], and a feasibility assessment was included for each topic (R28,R29). Justifications for considering feasibility mentioned were:

“So, they’re [WHO] very much focused on safety and clinical trials on efficacy. But at the end, when you have real life studies, they, they tend to balance the discussion.” (R30)

There were diverging views on, i) whether contextual evidence should be included in global guidelines development and if it is included, ii) what kind of contextual evidence is needed and iii) how contextual evidence should be assessed. Several external stakeholders thought that the WHO should not review contextual evidence when making global guidelines; it was felt that these reviews should be left to other stakeholders and countries’ appraisal when translating global recommendations into national antimalarial policy to avoid delaying releasing global recommendations (R24, R25, R32).

“…there’s sometimes a lengthy procedure, sometimes that long time. Sometimes we prefer they [WHO] make some quicker recommendation and sometimes the recommendation is not very clear to the country.” (R36)

WHO respondents’ views differed on whether issuing broader guidelines that could be adapted by countries to suit their contexts or continuing to review context in global policy decision-making was best:

“…now the entire policy development process is being reviewed, all policies are being revisited, so that people do not confuse what is the actual evidence-based policy recommendation, to the best practice guidance that the WHO attaches to that policy. And that best practice guidance is really flexible and should be adapted to the context”. (R19)

“I would say that even if in the…decision table [GRADE], there is like feasibility, acceptability, they are there however, there is not any requirement to have those studies as a precondition to then deliver the medicines or the vaccine or the diagnostics. Recently, we had this strong internal debates, for instance, for tafenoquine and point of care, this expanded test, whether we need some implementation studies to make recommendations and we were strongly divided internally definitely.” (R13)

“…this is part of the process for every guideline development group, they have to go through this formality. So, this is not an option. This is part of the decision-making [looking at feasibility]” (R13).

Several respondents said that the Cochrane system, designed to systematically assess RCT evidence, is unsuitable as a framework for assessing contextual evidence (R12, R13, R28). Currently, there are no defined guidelines for assessing these types of studies (R31). Lack of a suitable framework for assessment makes it unclear how exactly these studies *inform* final recommendations and suggests that composition and power dynamics of decision-making groups may impact how these studies are reviewed [32].

“It is looked at but it’s a really hard one because feasibility honestly, and this is my personal view, I would either delete it or be incredibly stringent as to what I mean by feasibility. So, because feasibility is time and space dependent, the feasibility of one intervention in Indonesia, of implementing a given intervention in Indonesia with a reasonably well-developed health system, and financial capacity may be or is I’m sure it’s very different to the Central African Republic.” (R27)

Estimating how much evidence is sufficient to develop a guideline is also complex and depends on many factors including an organisation’s or group’s individual risk aversion, understanding of new tools and, assessment of the strength and validity of available evidence [49, 50] (R12, R28, R31). Therefore, GDGs are not provided with benchmarks of required evidence instead relying on expert views to gauge how much evidence is sufficient to make a recommendation in consultation with the GMP (R13, R27, R28).

WHO stipulates that a recommendation should be worded as strong or conditional based on evidence quality, values & preferences, balance of benefits & harms, resource implications, priority of the problem, equity & human rights, acceptability, and feasibility [27]. There is also a distinction between the WHO’s “evidence-based policy recommendations” used by countries to revise national treatment guidelines and its “best-practice guidance” (often shortened to “guidance”) to countries (R19). *Guidance* was defined as flexible technical advice provided to countries on translating WHO *recommendations* into national recommendations (R19). External respondents agreed with this approach:

“I think WHO should keep its recommendation simple. This is a 8 aminoquinoline they should say for the radical cure of vivax, here is one option that you can use. And by the way, in populations, where you have a certain percentage of G6PD deficiency, you should always do a G6PD test…The problem is that sometimes they want to be a bit too detailed directive, make it also about operational guidance, etc, etc. And I think countries are broadly able to decide that themselves.” (R24)

### Moderating effects of funding on decision-making processes

The context in which the GMP conducts its guideline development process is shaped by its funding model. While writing this paper, over 50% of GMP’s operational budget is funded through a philanthropic organisation (R24). The remainder of funding is mostly from World Health Assembly member states [51] but their funding to the WHO has remained static for several years (R10, R13, R24).

“…there’s always this tension of…the member states saying prioritise, be leaner, be faster, while at the same time adding more and more and more priorities and not adding resources.” (R34)

The GMP reports to its major donor on how it allocates resources with regular activity review meetings (R14). This donor also funds key staffing positions at GMP headquarters and regional WHO offices (R13, R14). Both WHO and external stakeholders (including those from donor organisations) stated that there is no direct influence on the GMP’s policy decisions by donors:

“…in my opinion, the last thing you want is one agency, whether it’s the Gates Foundation, or the US government, or whoever puts the money on the table, to drive the priorities of GMP. I think that would be completely wrong.” (R24)

However, one respondent described indirect ways that WHO policy decision-making could be shaped by donors:

“I think, one of the, one of the biggest challenges we’ll have, and this is not only to do with malaria… they say the…one who pays the piper tries to dictate the tune. You may not try to dictate the tune directly but if you give me a pool of money for specific projects, in a way, you are defining the tune.” (R14)

Excessive donor influence on the GMP’s decision-making is avoided by obtaining the MPAG’s advice that acts as a filtering mechanism and a balanced advisory group to the GMP (R27, R34).

Several external stakeholders noted that the continuing decline in member state funding of the WHO necessitates the GMP’s increasing dependence on alternative donors (R23, R24, R32):

“So, there’s this real mismatch between what member states want them [WHO] to be empowered to do, but what they’re willing to give them the money for.” (R32)

Some of the GMP’s donors also invest upstream in new tools and research which can make the global policy change process quicker as the WHO is then collecting the right data in the right format for policy decision-making.

### Process transparency and communication

There were divergent views on the importance and benefits of increased transparency and the need to balance this with request for confidentiality in the decision-making process. GDG members cannot disclose meeting proceedings while a recommendation is in development as the information discussed at GDG meetings is complex and confidential until a recommendation is finalised (R13, R27, R29, R33). Some decision-makers suggested that tabling GDG meeting minutes as done for MPAG meetings could improve transparency of decision-making (R13, R28). Some also (R13, R28, R30) conceded that current closed-door proceedings for finalising recommendations are not ideal for public scrutiny of any conflicts of interest (COIs) and that these final steps should be made “more transparent” perhaps even “recorded” and made available publicly:

“…I think many agencies…which have a regulatory normative function are moving into this full transparency or on request, the public may access, the disclosure of the minutes of the meetings.” (R13)

Others however highlighted those final closed-door deliberations ensured confidential and open discussions within these groups, protected decisionmakers’ identity and enabled the GMP to arrive at appropriate recommendations without undue “external pressure” (R14, R29). Most external stakeholders however felt that closed-door proceedings made it difficult to understand how a final recommendation is made (R2, R4, R6, R7, R24, R26, R32).

The GMP’s current public communication strategy is to present “living guidelines”, with individual recommendations updated as needed, in keeping with WHO-wide public communication strategy:

“Living guidelines, essentially, it’s all the [WHO] guidelines into one.” (R19)

“…in the past, we, we used to do every five, six years a big review of the treatment guidelines, where basically you will, you come to a new edition, everything is updated…now, under the process of living guidelines, you don’t do a systematic review of the whole document. But you will only update single recommendations…And you will click and only get the last version with the last recommendation already included.” (R13)

Most respondents considered this approach an improvement on the previous approach of publishing a full set of guidelines every few years (R11, R13, R19, R27, R33). To increase public awareness of processes and decision-making, the GMP also hosts webinars to update external stakeholders on current GMP guideline recommendation priorities and MPAG proceedings. However, questions raised by participants are moderated so only queries that the host selects are publicly displayed during these webinars (R26). This was also viewed by external stakeholders as shrouding transparency of proceedings and discouraging open debate among stakeholders.

## Discussion

This study explored how evidence is adopted into policy by the GMP, what other factors impact policy, and if this process delivers timely guidance for countries. We interviewed stakeholders over two years to understand these factors. The following themes emerged from the data. Firstly, results indicate that while there are clearly defined ‘triggers’ for the new WHO prequalification and policy recommendation processes, these triggers did not immediately initiate these processes for *P*. *vivax* malaria in recent years. Secondly, while a rigorous and documented evidence review system is in place, including multiple checks and balances to ensure impartial recommendations, prioritisation within the GMP can influence timeliness of the development process. Thirdly, evidence types assessed during the process are geared toward highest quality evidence. However, contextual evidence is often included in review processes without a clear definition on how that evidence should be assessed. This means that power dynamics in decision-making groups may influence the process. Finally, some respondents perceive the current funding model for GMP activities to have indirect effect on which policies are prioritised and, most respondents felt that greater transparency on the final steps in the global policy process would benefit all stakeholders.

Over the last five years, there have been several potential triggers for updating guidelines for treating *P*. *vivax*, such as emergence of new evidence and registration of new drugs and diagnostic tools by SRAs. The first was submission of the adult tafenoquine dossier that was approved by the U.S. Food & Drug Administration (US FDA) in 2018 and thereafter submitted to and approved by the Australian Therapeutic Goods Administration (TGA) in 2019 [52]. Secondly, registration of a quantitative G6PD analyser was submitted to the TGA in 2021 (personal communication regarding SDBiosensor). And thirdly two large trials on high dose short course primaquine with more than 2000 patients were completed and published in 2019 [22, 23] and a further study published in 2022 [53].

Respondents identified these events as potential triggers, yet the first two have not yet initiated a policy change process while the third took almost three years to be incorporated into guidelines. Previously, even longer delays in updating global guidelines for malaria in pregnancy have also been criticised by external stakeholders [54].

From our study, the most likely reason for this lack of initiation was the complexity [55, 56] of the recommendations required including the need to coordinate with the prequalification unit for tafenoquine because of requirement for its use with an appropriate near patient G6PD test (as per the drug manufacturer’s instructions). COVID related delays for policy change were also mentioned by respondents; similar delays and loss of priority and progress in malaria interventions due to COVID have been identified in Africa [57]. Other potential reasons identified from our study and other literature are organisational resistance to change (including policy decision-makers’ leadership and prioritisation approaches) and need for an appropriate opportunity to change policy (including donor support) [55, 56, 58, 59]. Experience from tuberculosis and HIV drug approvals shows that time from publication of peer-reviewed evidence to SRA approval of drugs (before policy uptake) varied between 2.6 to 19+ years [60], highlighting that the extended timelines for uptake into policy are perhaps more norm than the exception. Understanding these significant variations in evidence uptake timelines means acknowledging that policy decision-making is multi-faceted and not only dependent on evidence; it also depends on expertise to inform judgements on evidence credibility and stakeholder views on how best to weigh trade-offs of competing policy outcomes and the perceived urgency of reaching a policy decision [61–64].

Prioritisation of global malaria policy change depends on decision-makers’ perception of the urgency of updating guidelines. Unclear mechanisms for priority setting allow for individual stakeholders’ priorities to have an increased weight. Political economy analysis and stakeholder analysis suggests that this is because hierarchies of authority and individual power dynamics influences policy agenda setting and policy design [58, 65, 66]. Approaches recommended for mitigating influence of individual stakeholders in setting policy agenda include ensuring that readily accessible standardised decision-making protocols are available for leaders when making decisions and, all relevant staff and external stakeholders are consulted when setting policy priorities [67]. WHO respondents estimated timelines for making recommendations as six to 18 months while external respondents suggested that this process can take up to 7–8 years (from experience with malaria in pregnancy recommendations). Many external stakeholders therefore think that global malaria policy recommendation processes are too slow for countries to adopt new tools quickly. When countries are waiting long periods for global malaria guidelines to be updated, it is difficult for them to plan their three-yearly funding applications to the Global Fund for procurement of drugs and diagnostics as the Global Fund usually only funds WHO approved tools [68].

As for all health policy decision-making, there is a hierarchy of evidence considered when making global malaria policy decisions. ‘Gold standard’ [61] evidence from RCTs is first considered, secondly evidence from cohort and observational studies and finally evidence from any available contextual studies [62]. Whether evidence types placed lower down in the hierarchy of quality of evidence such as cost-effectiveness, feasibility and implementation evidence require review at global or national policy level remains contentious [63, 64]. If contextual evidence is assessed at global level, more time may be needed for decision-making as such studies are often done after clinical studies. Adopting appropriate frameworks for assessing contextual evidence is important to, a) ensure that such evidence is reviewed systematically [69] and b) moderate individual decision-makers’ views on contextual evidence unduly shaping the review process [2, 5, 70, 71]. Alternatively, focusing global guidelines only on safety and efficacy evidence aligns better with the GMP’s role as a global advisory body enabling more country ownership of tailoring guidelines to specific contexts [72]. Even without a WHO recommendation, Brazil recently became the first country to adopt tafenoquine and the quantitative G6PD test for treating *P*. *vivax* relapse [73] after an evidence review by local and international stakeholders requested by its Ministry of Health and likewise Thailand [74]. The WHO recently launched a TB research tracker platform that tracks development of new treatment regimens, vaccines, and operational research projects aimed at improving TB prevention, treatment, and care [75]. It could consider doing likewise for malaria to expedite uptake of evidence into policy.

Rise of non-state and non-United Nations actors such as the Global Fund, GAVI, the Gates Foundation and the World Bank’s roles in global health funding and governance has changed the status quo largely positively but it also leans global health policymaking priorities towards specific interests and ideas of these organisations [76]. Our findings align with this appraisal of the role of non-state actors in global health currently. Respondents expressed reservations regarding how non-state donors may indirectly shape the GMP’s priorities and activities and thereby global malaria guideline decision-making through malaria initiatives that they fund. Continuing decline in member state funding to the WHO exacerbates this situation [76]. However, the success of global health policy initiatives often depends on availability of targeted funding that can only be provided by specific, well-endowed donors [77, 78]. Donor influence is wielded indirectly by malaria interventions that a donor supports [79, 80]. Whether the GMP’s aims and policy priorities and its relationship with donors would differ if its funding model changed is unclear. Currently the WHO is working with Member States to find ways to improve WHO’s funding and ensure it is flexible, predictable and sustainable so that it can be more agile and strategic in its activities [51].

Finally, more transparent policy processes would create greater confidence in the GMP’s global recommendations by external stakeholders; noted the GMP’s 2022 updated malaria guidelines do provide more transparency on these processes than previously [81]. Further transparency on what actually triggers the policy process and on final closed-door decision-making processes ensures that best-practice principles of equity in decision-making are adhered to by all actors [1]. Improving the culture of transparency and collaborative knowledge translation between large institutions like the WHO and its external stakeholders takes time [82, 83]. Taking incremental steps to increase transparency of decision-making in consultation with external stakeholders (regarding what information they need) is one way to do this [84]. One such incremental improvement in process transparency and public communication is the MagicApp that currently publishes “living guidelines” compared to previous methods of publishing updated guidelines every five years. Further strategies to improve transparency of global malaria decision-making processes are needed for greater accountability to external stakeholders on decision-making [85].

Our analysis has several limitations. Firstly, it is based on stakeholders’ views on evolving global malaria policymaking processes. Interviews were conducted over two years during which time some WHO processes changed and WHO guidelines for *P*. *vivax* treatment were updated. Multiple respondents were interviewed twice or more. Secondly, the study focused on the global malaria policymaking environment only. However, understanding translation of these global processes into national guidelines is crucial for achieving desired impacts and is therefore subject to ongoing work. Further comparative analysis of other multilateral organisations’ policymaking processes may also be useful for understanding how to optimise global malaria policymaking.

## Conclusions

Our findings suggest a need to address five strategies to optimise global malaria policymaking. Firstly, ensure that identified triggers actually initiate the policy change process and make these triggers known to external stakeholders. Secondly, expedite decision-making timelines by developing a priority framework that could be used to guide decisions on which recommendations to prioritise (as new evidence emerges) and publicise this framework. Thirdly, adopt suitable frameworks for assessing contextual evidence. Fourth, work with WHO member states to ensure that non-state funders have a well moderated and transparent role in any organisational or policymaking processes. Lastly, increase transparency of policy decision-making processes when publishing new recommendations.

## Supporting information

S1 Appendix Synthesis of adopted frameworks. (DOCX)

S2 ppendix List of reviewed documents. (DOCX)

S3 Appendix High-level diagram of the Global Malaria Programme’s policy pathway for new products. (DOCX)

S4 Appendix Overview of the process of guideline development. (DOCX)

S5 Appendix Declaration of interest form by senior WHO staff. (DOCX)

S6 Appendix Guideline development group member composition. (DOCX)

S7 Appendix Key changes made to GMP processes after 2018. (DOCX)
