# Supplementary material for: Evidence uptake is only part of the process: Stakeholders’ insights on WHO treatment guideline recommendation processes for radical cure of P. vivax malaria
Source: PLOS Glob Public Health. 2024 Mar 14;4(3):e0002990. doi: 10.1371/journal.pgph.0002990 (PMC10939226; doi:10.1371/journal.pgph.0002990)
Supplement: S1 Appendix — (DOCX) [file pgph.0002990.s001.docx]

**Appendix 1**

**Synthesis of adopted frameworks**

| **Category** | **Category description** | **Barriers & Enablers relating to category** | **Description of barriers and enablers** |
| --- | --- | --- | --- |
| Interests (3is) | Actors’ agenda – how they *frame* a policy issue (based on their expert knowledge). | Organisational  Individual | Organisational interests of the WHO and of external malaria stakeholders.  How individual interests of malaria stakeholders may shape policy decisions. |
| Ideas (3is & Shiffman) | Ways in which those involved in an issue understand and portray it. | Individual | How individual ideas of malaria stakeholders may shape policy decisions. |
| Institutions (3is) | Organisations that influence global malaria policy. | Organisational: processes, policy and public communication approach  Timing of recommendations | WHO processes, policies and communication strategy: new streamlined processes between the GMP and the prequalification unit; GMP’s guideline development processes; overall WHO organisational aims and processes; public communication strategy (e.g., MagicApp) and timing of making recommendations. |
| Actor power  (Shiffman) | Ability to act on policy. | Organisational hierarchy and processes, resources and, available evidence | Actors’ position within the organisation & decision-making autonomy.  Availability of appropriate resources and autonomy to use resources.  Evidence: new or emerging on *P. vivax* malaria radical cure options. |
| Context (Shiffman) | Wider contextual environment in which actors operate. | Policymaking context | Countries’ needs and the WHO’s perception of global disease burden of *P. vivax* malaria. How do country objectives and the WHO’s objectives align. |
| Issue characteristics  (Shiffman) | Features of the problem being addressed. | Disease burden and morbidity | The WHO’s and other malaria stakeholders’ perception of *P. vivax* global disease burden and morbidity. |
