# Supplementary material for: Evidence uptake is only part of the process: Stakeholders’ insights on WHO treatment guideline recommendation processes for radical cure of P. vivax malaria
Source: PLOS Glob Public Health. 2024 Mar 14;4(3):e0002990. doi: 10.1371/journal.pgph.0002990 (PMC10939226; doi:10.1371/journal.pgph.0002990)
Supplement: S2 ppendix — (DOCX) [file pgph.0002990.s002.docx]

**Appendix 2**

**List of reviewed documents**

| Type of document | Number of documents |
| --- | --- |
| WHO policy guidelines, recommendations & strategic plans | 6 |
| Email correspondence with WHO respondents | 4 |
| Public communications (websites, factsheets) | 3 |
| Internal WHO document | 1 |
