# Supplementary material for: Evidence uptake is only part of the process: Stakeholders’ insights on WHO treatment guideline recommendation processes for radical cure of P. vivax malaria
Source: PLOS Glob Public Health. 2024 Mar 14;4(3):e0002990. doi: 10.1371/journal.pgph.0002990 (PMC10939226; doi:10.1371/journal.pgph.0002990)
Supplement: S3 Appendix — (DOCX) [file pgph.0002990.s003.docx]

**Appendix 3**


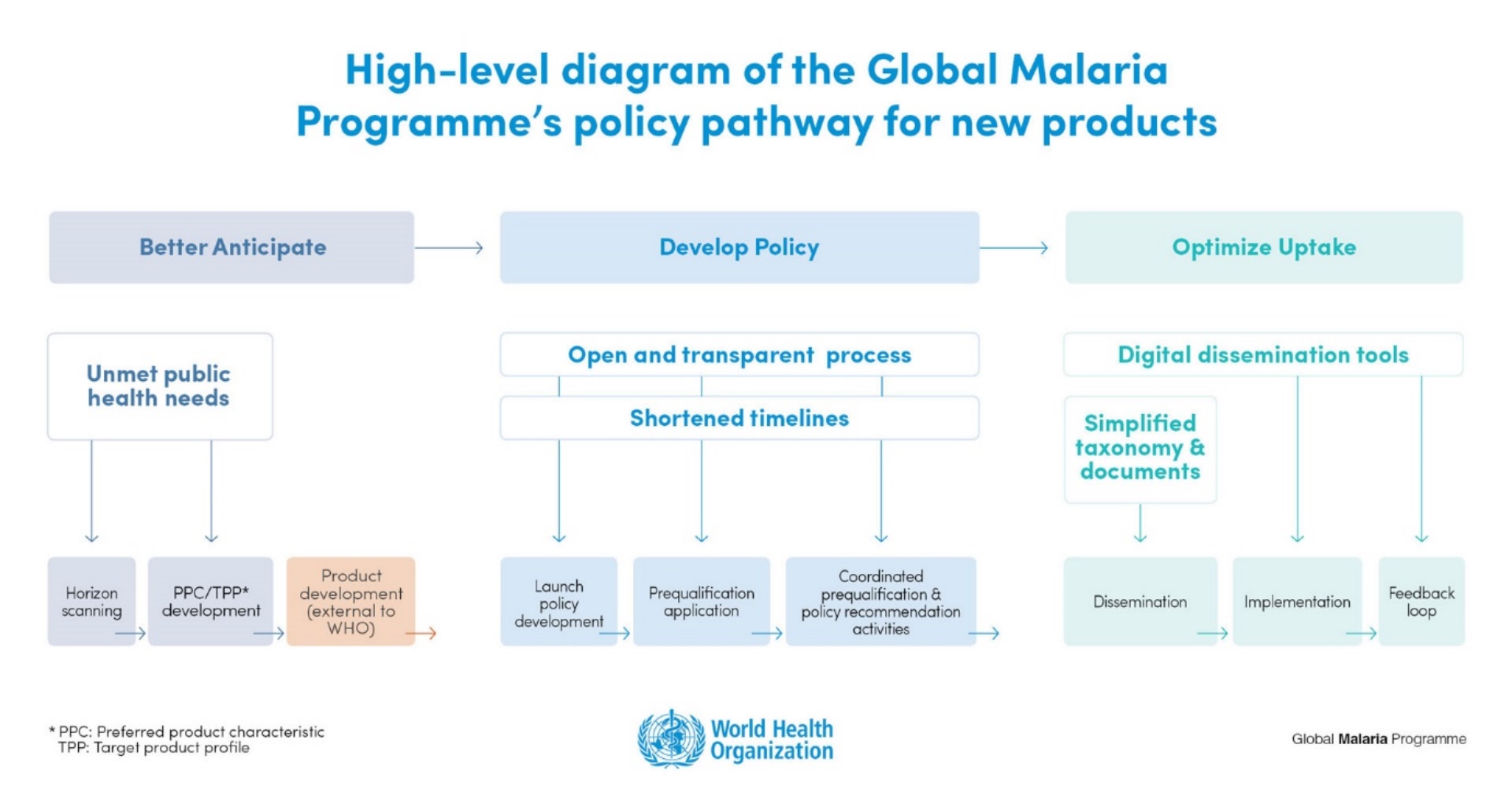


*PPC: Preferred product characteristic *TPP: Target product profile

Source: WHO. High level diagram of the GMP policy pathway. Geneva: World Health Organization; 2020 [cited 2020 01/10/2020] [1].

[Terms of use of the WHO website](https://www.who.int/about/policies/terms-of-use): https://www.who.int/about/policies/terms-of-use
