# Supplementary material for: Evidence uptake is only part of the process: Stakeholders’ insights on WHO treatment guideline recommendation processes for radical cure of P. vivax malaria
Source: PLOS Glob Public Health. 2024 Mar 14;4(3):e0002990. doi: 10.1371/journal.pgph.0002990 (PMC10939226; doi:10.1371/journal.pgph.0002990)
Supplement: S4 Appendix — (DOCX) [file pgph.0002990.s004.docx]

**Appendix 4**


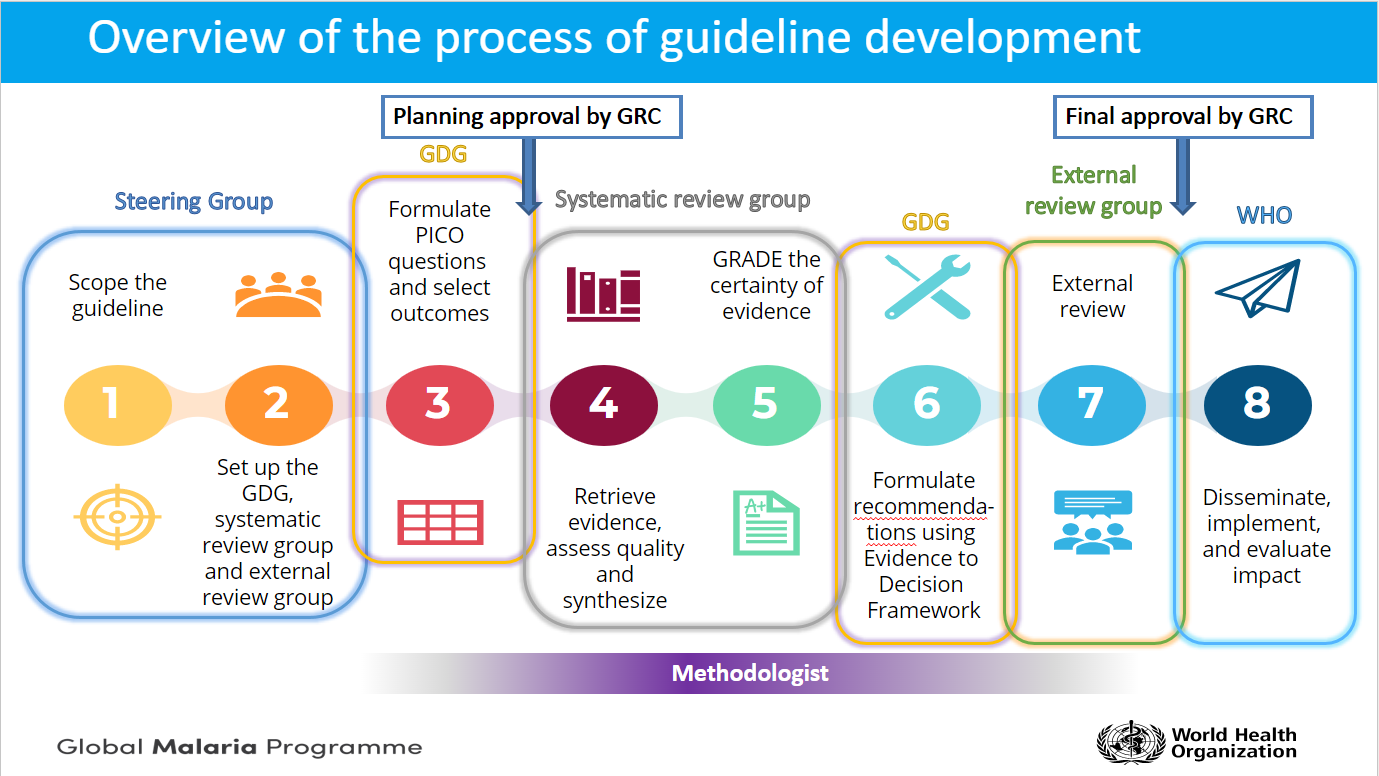


Source: Lindblade K, editor WHO Guideline Development Process. Workshop on WHO policy guidance on malaria elimination and the implementation of the intensification plans for reducing malaria burden in the Greater Mekong Subregion; 2021 22/11/21; Virtual meeting: World Health Organization [1].

Terms of use: https://www.who.int/about/policies/terms-of-use
