# Supplementary material for: Evidence uptake is only part of the process: Stakeholders’ insights on WHO treatment guideline recommendation processes for radical cure of P. vivax malaria
Source: PLOS Glob Public Health. 2024 Mar 14;4(3):e0002990. doi: 10.1371/journal.pgph.0002990 (PMC10939226; doi:10.1371/journal.pgph.0002990)
Supplement: S5 Appendix — (DOCX) [file pgph.0002990.s005.docx]

**Appendix 5**

**Declaration of Interest form by senior WHO staff**

DECLARATION OF INTERESTS FOR WHO EXPERTS WHO's work on global health issues requires the assistance of external experts who may have interests related to their expertise. To ensure the highest integrity and public confidence in its activities, WHO requires that experts serving in an advisory role disclose any circumstances that could give rise to a potential conflict of interest related to the subject of the activity in which they will be involved. All experts serving in an advisory role must disclose any circumstances that could represent a potential conflict of interest (i.e., any interest that may affect, or may reasonably be perceived to affect, the expert's objectivity and independence). You must disclose on this Declaration of Interest (DOI) form any financial, professional or other interest relevant to the subject of the work or meeting in which you have been asked to participate in or contribute towards and any interest that could be affected by the outcome of the meeting or work. You must also declare relevant interests of your immediate family members (see definition below) and, if you are aware of it, relevant interests of other parties with whom you have substantial common interests and which may be perceived as unduly influencing your judgement (e.g., employer, close professional associates, administrative unit or department). Please note that not fully completing and disclosing all relevant information on this form may, depending on the circumstances, lead WHO to decide not to appoint you to WHO advisory bodies/functions in the future. Please complete this form and submit it to WHO Secretariat if possible at least 4 weeks but no later than 2 weeks before the meeting or work. You must also promptly inform the Secretariat if there is any change in this information prior to, or during the course of, the meeting or work. All experts must complete this form before participation in a WHO activity can be confirmed. Please note that not fully completing and disclosing all relevant information on this form may, depending on the circumstances, lead WHO to decide not to appoint you to WHO advisory bodies/functions in the future. Answering "Yes" to a question on this form does not automatically disqualify you or limit your participation in a WHO activity. Your answers will be reviewed by the Secretariat to determine whether you have a conflict of interest relevant to the subject at hand. One of the outcomes listed in the next paragraph can occur depending on the circumstances (e.g., nature and magnitude of the interest, timeframe and duration of the interest). The Secretariat may conclude that no potential conflict exists or that the interest is irrelevant or insignificant. If, however, a declared interest is determined to be potentially or clearly significant, one or more of the following three measures for managing the conflict of interest may be applied. The Secretariat (i) allows full participation, with public disclosure of your interest; (ii) mandates partial exclusion (i.e., you will be excluded from that portion of the meeting or work related to the declared interest and from the corresponding decision-making process); or (iii) mandates total exclusion (i.e., you will not be able to participate in any part of the meeting or work). All potentially significant interests will be disclosed to the other participants at the start of the activity and you will be asked if there have been any changes. A summary of all declarations and actions taken to manage any declared interests will be published in resulting reports and work products. Furthermore, if the objectivity of the work or meeting in which you are involved is subsequently questioned, the contents of your DOI form may be made available by the Secretariat to persons outside WHO if the Director-General considers such disclosure to be in the best interest of the Organization, after consulting with you. Completing this DOI form means that you agree to these conditions. If you are unable or unwilling to disclose the details of an interest that may pose a real or perceived conflict, you must disclose that a conflict of interest may exist and the Secretariat may decide that you be totally recused from the meeting or work concerned, after consulting with you.

Name:

Institution:

Email: Date and title of meeting or work, including description of subject matter to be considered (if a number of substances or processes are to be evaluated, a list should be attached by the organizer of the activity):

Please answer each of the questions below. If the answer to any of the questions is "yes", briefly describe the circumstances on the last page of the form. The term "you" refers to yourself and your immediate family members (i.e., spouse (or partner with whom you have a similar close personal relationship) and your children). "Commercial entity" includes any commercial business, an industry association, research institution or other enterprise whose funding is significantly derived from commercial sources with an interest related to the subject of the meeting or work. "Organization" includes a governmental, international or non-profit organization. "Meeting" includes a series or cycle of meetings.

EMPLOYMENT AND CONSULTING Within the past 4 years, have you received remuneration from a commercial entity or other organization with an interest related to the subject of the meeting or work?

1a Employment Yes No

1b Consulting, including service as a technical or other advisor Yes No

RESEARCH SUPPORT Within the past 4 years, have you or has your research unit received support from a commercial entity or other organization with an interest related to the subject of the meeting or work?

2a Research support, including grants, collaborations, sponsorships, and other funding Yes No

2b Non-monetary support valued at more than US $1000 overall (include equipment, facilities, research assistants, paid travel to meetings, etc.) Support (including honoraria) for being on a speakers bureau, giving speeches or training for a commercial entity or other organization with an interest related to the subject of the meeting or work? Yes No

INVESTMENT INTERESTS Do you have current investments (valued at more than US $5 000 overall) in a commercial entity with an interest related to the subject of the meeting or work? Please also include indirect investments such as a trust or holding company. You may exclude mutual funds, pension funds or similar investments that are broadly diversified and on which you exercise no control.

3a Stocks, bonds, stock options, other securities (e.g., short sales) Yes No

3b Commercial business interests (e.g., proprietorships, partnerships, joint ventures, board memberships, controlling interest in a company) Yes No

INTELLECTUAL PROPERTY Do you have any intellectual property rights that might be enhanced or diminished by the outcome of the meeting or work?

4a Patents, trademarks, or copyrights (including pending applications) Yes No

4b Proprietary know-how in a substance, technology or process Yes No

PUBLIC STATEMENTS AND POSITIONS (during the past 3 years)

5a As part of a regulatory, legislative or judicial process, have you provided an expert opinion or testimony, related to the subject of the meeting or work, for a commercial entity or other organization? Yes No

5b Have you held an office or other position, paid or unpaid, where you represented interests or defended a position related to the subject of the meeting or work? Yes No

ADDITIONAL INFORMATION

6a If not already disclosed above, have you worked for the competitor of a product that is the subject of the meeting or work, or will your participation in the meeting or work enable you to obtain access to a competitor's confidential proprietary information, or create for you a personal, professional, financial or business competitive advantage? Yes No

6b To your knowledge, would the outcome of the meeting or work benefit or adversely affect interests of others with whom you have substantial common personal, professional, financial or business interests (such as your adult children or siblings, close professional colleagues, administrative unit or department)? Yes No

6c Excluding WHO, has any person or entity paid or contributed towards your travel costs in connection with this WHO meeting or work? Yes No

6d Have you received any payments (other than for travel costs) or honoraria for speaking publicly on the subject of this WHO meeting or work? Yes No

6e Is there any other aspect of your background or present circumstances not addressed above that might be perceived as affecting your objectivity or independence? Yes No

7.

TOBACCO OR TOBACCO PRODUCTS (answer without regard to relevance to the subject of the meeting or work) Within the past 4 years, have you had employment or received research support or other funding from, or had any other professional relationship with, an entity directly involved in the production, manufacture, distribution or sale of tobacco or tobacco products or representing the interests of any such entity? Yes No EXPLANATION OF "YES" RESPONSES: If the answer to any of the above questions is "yes", check above and briefly describe the circumstances on this page. If you do not describe the nature of an interest or if you do not provide the amount or value involved where relevant, the conflict will be assumed to be significant.

Nos. 1 - 4: Type of interest, question number and category (e.g., Intellectual Property 4.a copyrights) and basic descriptive details. Name of company, organization, or institution Belongs to you, a family member, employer, research unit or other? Amount of income or value of interest (if not disclosed, is assumed to be significant) Current interest (or year ceased)

Nos. 5-6: Describe the subject, specific circumstances, parties involved, time frame and other relevant details

CONSENT TO DISCLOSURE. By completing and signing this form, you consent to the disclosure of any relevant conflicts to other meeting participants and in the resulting report or work product. DECLARATION. I hereby declare on my honour that the disclosed information is true and complete to the best of my knowledge. Should there be any change to the above information, I will promptly notify the responsible staff of WHO and complete a new declaration of interest form that describes the changes. This includes any change that occurs before or during the meeting or work itself and through the period up to the publication of the final results or completion of the activity concerned. Date: ________________ Signature_____________________
