# Supplementary material for: Evidence uptake is only part of the process: Stakeholders’ insights on WHO treatment guideline recommendation processes for radical cure of P. vivax malaria
Source: PLOS Glob Public Health. 2024 Mar 14;4(3):e0002990. doi: 10.1371/journal.pgph.0002990 (PMC10939226; doi:10.1371/journal.pgph.0002990)
Supplement: S6 Appendix — (DOCX) [file pgph.0002990.s006.docx]

**Appendix 6**

**Guideline Development Group member composition (as per WHO handbook for guideline development[1])**

| Relevant technical experts |
| --- |
| End-users, such as programme managers and health professionals, who will adopt, adapt, and implement the guideline |
| Representatives of groups most affected by the recommendations in the guideline, such as service users and representatives of disadvantaged groups |
| Experts in assessing evidence and developing guidelines informed by evidence |
| Other technical experts as required (e.g., a health economist or an expert on equity, human rights and gender) |

- Usually, a GDG comprises of 10-20 members although it may occasionally have more members.
- Selection of a GDG chair is a key decision made by the Guideline Steering Group, but the choice is generally agreed on by all GDG members.
