# Supplementary material for: Evidence uptake is only part of the process: Stakeholders’ insights on WHO treatment guideline recommendation processes for radical cure of P. vivax malaria
Source: PLOS Glob Public Health. 2024 Mar 14;4(3):e0002990. doi: 10.1371/journal.pgph.0002990 (PMC10939226; doi:10.1371/journal.pgph.0002990)
Supplement: S7 Appendix — (DOCX) [file pgph.0002990.s007.docx]

**Appendix 7**

**Key changes made to GMP processes after 2018**

Several changes were made to make malaria policy recommendation processes more efficient since 2018. Most significantly, streamlined organisational processes between the GMP and the prequalification unit to ensure coordinated delivery of global malaria guidelines and prequalification of new malaria drugs and diagnostics. Previously, standard practice was for the GMP to make a guideline and then for the WHO prequalification pipeline to be opened for approving any new tools needed, resulting in a time lag between these two processes. However, instances when the prequalification unit approved a drug that the GMP had not yet approved also occurred, again reflecting time lags between these two processes. Changes to improve interdepartmental communication between the GMP and the prequalification unit, both formal and informal, have also been established.

Other key changes in the GMP’s policy processes are more reliance on GDGs for decision-making and the introduction of an Editorial Group to review the wording of GMP’s guidelines before publication. To standardise guideline development processes across all WHO departments, the GMP now relies on GDGs to refine PICO questions, oversee systematic reviews of evidence, and makes. GDGs are considered “the engine of the guideline development process” (R#12). GDGs should include external malaria stakeholders with good knowledge of the topic under discussion including malaria researchers, national malaria programme (NMP) representatives, experts in human rights, civil society and social equity, and a methodologist (appendix 6). The newly introduced Editorial Group − convenes towards the end of the guideline development process to ensure consistency and clarity in the wording of all GMP guidelines. While writing this paper, the GMP was the only WHO disease department employing an Editorial Group in its policy recommendation process. Since 2020, the WHO’s Department of Quality Assurance, Norms and Standards also provides guidance and oversight to all WHO disease departments on making best-practice policy decisions [1]. This division is seen as more rigorous in, “all the phases of initial review, planning of the proposal, approval of the proposal, review of the results…the multiple directors and EDGs [executive decision groups] which need to sign off” before a recommendation is made (R#13, R#27).

While these changes to policymaking processes aim to improve and standardise guideline development across the WHO, these could also extend time needed to make new guidelines. The WHO handbook for guideline development [2] and current policy process diagram that the GMP presents at public webinars [3] provide clear overviews of its processes yet, *internal* organisational processes on how coordination between the GMP and the prequalification unit, and any other related WHO departments takes place remain opaque to many external stakeholders. There were different opinions within the WHO on the GMP’s new policy decision-making processes although most WHO respondents thought that these changes will eventually improve policy recommendation processes. One WHO respondent said that the overhaul did not acknowledge considerable efforts made by the GMP before 2018 to improve their policymaking processes. Another emphasised that “we are not a monolithic organisation” and therefore some flexibility regarding new standardised WHO policy processes was needed to make guidelines (R#14).

1. WHO. Guiding for impact in a rapidly changing world. WHO Department of Quality Assurance, Norms and Standards (QNS): 2020-2021 in review. Geneva: World Health Organization, 2022 Contract No.: 25/07/2022.

2. WHO. WHO Handbook for Guideline Development. 2nd ed. Geneva: World Health Organization; 2014.

3. Lindblade K, editor WHO Guideline Development Process. Workshop on WHO policy guidance on malaria elimination and the implementation of the intensification plans for reducing malaria burden in the Greater Mekong Subregion; 2021 22/11/21; Virtual meeting: World Health Organization.
